# Editorial: Exploring Gender and Sex Differences in Behavioral Dyscontrol: From Drug Addiction to Impulse Control Disorders

**DOI:** 10.3389/fpsyt.2016.00019

**Published:** 2016-02-22

**Authors:** Liana Fattore, Miriam Melis

**Affiliations:** ^1^CNR Neuroscience Institute, National Research Council, Cagliari, Italy; ^2^Department of Biomedical Sciences, University of Cagliari, Cagliari, Italy

**Keywords:** gender differences, sex differences, behavioral addictions, drug addiction, impulse control disorders, sex hormones, sexual brain dimorphism

**The Editorial on the Research Topic**

**Exploring Gender and Sex Differences in Behavioral Dyscontrol: From Drug Addiction to Impulse Control Disorders**

This Research Topic gathers animal and human research articles, reviews, and opinion articles on current research in the field of sex and gender differences in behavioral addictions, which are complex disorders with interacting factors, including environmental factors, comorbidity, and personality traits (1, 2). Research in this field adds to the long-held argument that male and female brains do differ. However, while evidence exists on how male and female brains functionally differ (3–5), conflicting findings suggest that human brains cannot be narrowly classified into sexes/genders (6).

Over the past decade, there have been major advances in our understanding of sex and gender differences in behavioral addictions and underlying motivations (7, 8), brain reward processes (9), and impulsive behaviors (10). Potential factors, which could provide a neurobiological basis for sex- and gender-based differences in behavioral addictions, have been identified. Among them, there are organizational and activational effects of gonadal hormones, socio-cultural factors, different impulse-control ability, and responsiveness to stress (11, 12). It is important to note that, although often used as synonymous, the terms “sex” and “gender” are not interchangeable. In fact, the term “sex” is referred to biological attributes and characteristics associated with the adjectives “male” and “female” (i.e., anatomy and physiology inherent male-female differences), while “gender” concerns sociocultural distinctions between males and females (i.e., culture-related dogmas and roles, behaviors embraced by men and women that shape their daily life and activities).

In this Research Topic, basic researchers and clinicians that are leading experts in the field provide original findings and overviews on the role of sex and gender in modulating addictive behaviors and developing behavioral addictions. First, sex and gender differences in addiction to cannabis, methamphetamine, cocaine, and alcohol are discussed. Rubino and Parolaro systematically reviewed human and animal studies showing how males and females respond differently to cannabinoid compounds. In particular, dichotomy in the pharmacokinetics of THC observed in males and females may contribute to their dissimilar responses to cannabinoids. The role of sexual dimorphism in the brain endocannabinoid system and its interaction with gonadal hormones may also play a part (13–18). Accordingly, Ruda-Kucerova et al. presented evidence that sex-dependent differences exist in the reinstatement of methamphetamine-seeking behavior in abstinent rats. Notably, females displayed higher vulnerability to relapse to methamphetamine seeking independently of the current estrous cycle phase, thus suggesting that central mechanisms responsible for the enhanced response displayed by females are not affected by circulating ovarian hormones.

In humans, sex and gender differences are reported in psychiatric comorbidity and plasma biomarkers in abstinent cocaine addicts and in behavioral impulsivity in heavy alcohol drinkers. Specifically, Pedraz et al. demonstrated that cocaine addicted men and women differ with regard to the levels of plasma biomarkers for cocaine addiction. Notably, while men exhibit a higher incidence of substances use comorbidity (e.g., alcohol), cocaine addicted women display a higher prevalence of comorbid psychiatric disorders (e.g., anxiety). In addition, Weafer et al. demonstrated that heavy alcohol female drinkers show poorer inhibitory control than male drinkers do, although it remains to be determined whether the reported higher behavioral impulsivity in women is the cause or the consequence of heavy drinking. That behavioral control is impacted by sex and gender is further supported by evidence compellingly reviewed by Carroll and Smethells revealing sex- and gender-dependent differences in impulsivity, food, and drug addiction. Importantly, authors discussed pharmacological and behavioral treatments for improving control of impulses in a sex-tailored manner, an approach also suggested for drug and behavioral addictions (19, 20). Among behavioral addictions, sexual addiction (often referred to as compulsive sexual behavior) is discussed by Weinstein et al. who found that men are more likely to use cybersex and experience craving for pornography than women. Importantly, both craving for pornography and frequency of cybersex were associated with difficulty in forming intimate relationship. In another clinical setting, Davis et al. used a moderator-mediation model to perform an elegant analysis of personality risk-factors and sex in moderating the relationship between ADHD symptomatology and addictive behaviors. According to their observations, no sex differences in personality risk for addiction or in the use of addictive behaviors were found in ADHD patients. A positive affective neuroendocrinology (PANE) approach to study the mechanisms of reward motivation and dysregulation is herein proposed by Welker et al., which investigated sources of potential sex differences in the hormonal mechanisms of behavioral dyscontrol. Finally, Mitchell and Potenza highlighted the importance of investigating the relationship between sexual hormones and impulsivity traits to disentangle sex and gender differences in impulse control and behavioral addictions. Equally, Mendrek et al. emphasize the need of considering sex and gender in neuroscience by focusing on psychiatric disorders, such as schizophrenia and drug addiction, where research on such differences is still in its infancy.

We wish to thank all the Authors of this Research Topic for presenting and discussing their work and sharing their personal expertise and opinions on this emerging field. Moreover, we express gratitude to all reviewers that found time in their busy schedules to provide us with useful and constructive comments. We feel that research on factors and mechanisms allowing for the pursuit of drug and non-drug rewards is essential to deliver innovative gender-tailored treatments and to develop preventive strategies that may efficiently reduce in men and women the risk of becoming addicted to a substance or an activity. Improving our knowledge on sex and gender differences in drug addiction and reward processing will remarkably have therapeutic implications and help the development of sex-tailored, gender-sensitive treatment interventions. Some evidence has been already provided. In fact, pharmacological treatments differently affect male and female addicts. That is, the long-acting injectable form of naltrexone was found to be efficacious for males but not for females (21). This finding was further supported by the observation that oral naltrexone lacked efficacy relative to placebo in alcoholic women (22). Conversely, a 16-week course of fluoxetine initiated 8 weeks pre-quit cigarette smoking (“sequential” fluoxetine) reduced pre-quit depressive symptoms, withdrawal-relevant negative affect, and craving to smoke during a pre-quit period only in women (23). These and similar studies confirmed the importance of considering gender when examining treatment efficacy, and highlight the timeliness of this Research Topic.

## Author Contributions

LF and MM equally contributed to this Editorial for the Research Topic entitled: “Exploring gender and sex differences in behavioral dyscontrol: from drug addiction to impulse control disorders.”

## Conflict of Interest Statement

The authors declare that the research was conducted in the absence of any commercial or financial relationships that could be construed as a potential conflict of interest.
